# Long-term platelet priming after glycoprotein VI stimulation in comparison to Protease-Activating Receptor (PAR) stimulation

**DOI:** 10.1371/journal.pone.0247425

**Published:** 2021-03-03

**Authors:** Jinmi Zou, Jiayu Wu, Mark Roest, Johan W. M. Heemskerk

**Affiliations:** 1 Department of Biochemistry, CARIM, Maastricht University, Maastricht, The Netherlands; 2 Synapse Research Institute, Maastricht, The Netherlands; Ludwig-Maximilians-Universitat Munchen, GERMANY

## Abstract

Platelets can respond to multiple antagonists and agonists, implying that their activation state is a consequence of past exposure to these substances. While platelets are often considered as one-time responsive cells, they likely can respond to sequential application of inhibitors and stimuli. We hypothesized that the ability of platelets to sequentially respond depends on the time and type of repeated agonist application. The present proof-of-concept data show that iloprost (cAMP elevation), tirofiban (integrin α_IIb_β_3_ blocker) and Syk kinase inhibition subacutely modulated platelet aggregation, *i*.*e*. halted this process even when applied after agonist. In comparison to thrombin-activated receptor (PAR) stimulation, glycoprotein VI (GPVI) stimulation was less sensitive to time-dependent blockage of aggregation, with Syk inhibition as an exception. Furthermore, cytosolic Ca^2+^ measurements indicated that, when compared to PAR, prior GPVI stimulation induced a more persistent, priming activation state of platelets that influenced the response to a next agent. Overall, these data point to an unexpected priming memory of activated platelets in subacutely responding to another inhibitor or stimulus, with a higher versatility and faster offset after PAR stimulation than after GPVI stimulation.

## Introduction

Blood platelets are equipped with a broad range of adhesive and signalling receptors which synergize to trigger a common set of functional responses, in particular integrin activation, granular release and procoagulant activity [1, 2]. Studies with genetically modified mice have established that hundreds of genes encoding for platelet receptors, signalling molecules and granule components regulate the functions of platelets in physiological haemostasis and pathological arterial thrombosis [3]. The common concept herein is that platelet activation is suppressed by vessel wall-derived inhibitors, and that this suppression is relieved in the presence of a weak or strong agonist. With exceptions [4], most of the literature implicitly considers platelets as ’single activating’ cells, which idea supposes that after a first activation event the platelet response is ’over’ [1]. However, the circulating platelets will be continuously exposed to (ant)agonists, suggesting that they will experience moments of inactivation and activation, the balance of which can be altered under pathophysiological conditions [5, 6]. This notion implies that the inhibitory state of circulating platelets can be disturbed multiple times or, in other words, that platelets have the capacity over time to respond to series of agonists and antagonists. This has not been studied in detail before.

In the present paper, as a proof-of-concept, we tested the hypothesis that platelets can sequentially respond to more than one agonist and/or antagonist in a time-dependent manner. We therefore treated human platelets sequentially via: *(i)* the ITAM-linked collagen receptor glycoprotein VI (GPVI) using cross-linked collagen-related peptide (CRP-XL); *(ii)* the different pathway of G-protein-coupled receptors (GPCR) using the PAR1/4 agonist thrombin, the reversible PAR1 agonist TRAP6 [7], or the P2Y_1_/P2Y_12_ agonist Me-S-ADP; *(iii)* the platelet-inhibiting prostacyclin analogue, iloprost, elevating cAMP levels; and/or *(iv)* the integrin α_IIb_β_3_ antagonist tirofiban, suppressing platelet-fibrinogen interactions [2]. Our first research question was in which time frame platelet aggregation induced by an agonist can be suppressed by prior or later application of inhibitor. Our second question was in which time frame a first agonist can influence the platelet responses to a second agonist.

## Materials and methods

### Materials

Thrombin was obtained from Enzyme Research Laboratories (South Bend IN, USA). Thrombin receptor-activating peptide 6 (SFLLRN, TRAP6) was purchased from Bachem (Bubendorf, Switzerland); cross-linked collagen-related peptide (CRP-XL) was from the University of Cambridge (Cambridge, UK); the stable ADP analogue methylthio-adenosine-diphosphate (Me-S-ADP) [8] came from Santa Cruz Biotechnology (Dallas TX, USA). Fura-2 acetoxymethyl ester and human fibrinogen were obtained from Invitrogen (Carlsbad CA, USA); Pluronic F-127 from Molecular Probes (Eugene OR, USA). Integrin α_IIb_β_3_ inhibitor tirofiban [9] and cAMP-elevating agent iloprost [2] were from Sigma-Aldrich (St. Louis MI, USA); the selective Syk kinase inhibitor PRT-060318, 2-((1R,2S)-2-aminocyclohexylamino)-4-(m-tolylamino)pyrimidine-5-carboxamide (Syk-IN) [10] came from Bio-Connect (Huissen, The Netherlands).

### Blood collection

Human blood was collected from healthy volunteers, after full informed consent according to the Declaration of Helsinki. Approval for the studies was obtained from the local Medical Ethics Committee (METC 10-30-023, Maastricht University). The subjects had not used antiplatelet medication for at least 2 weeks. Venous blood was collected from an antecubital vein into 3.2% trisodium citrate Vacuette tubes (Greiner Bio-One, Alphen a/d Rijn, The Netherlands). The first tube of blood was discarded to avoid the presence of traces of tissue factor.

### Preparation of washed platelets

Platelet-rich plasma and washed platelets were prepared, basically as described before [9, 11]. In brief, blood samples were centrifuged at 190 *g* for 15 min (room temperature). The yellowish upper layer of PRP was carefully collected without taking the buffy coat or the red cell bottom layer. After addition of 10 vol% ACD medium (80 mM trisodium citrate, 52 mM citric acid and 180 mM glucose), platelets in the PRP were spin down in 2 mL Eppendorf tubes at 1700 *g* for 2 min. Plasma was removed and the tubes were held upside down for 1 min to remove remaining plasma traces. The pellets then were resuspended into 1 mL of Hepes buffer pH 6.6 (136 mM NaCl, 10 mM glucose, 5 mM Hepes, 2.7 mM KCl, 2 mM MgCl_2_, apyrase at 0.2 units ADPase/mL, and 0.1% (w/v) bovine serum albumin). After addition of 6.6 vol% ACD, the tubes were recentrifuged, and the washed pelleted platelets were finally resuspended into 1 mL Hepes buffer pH 7.45 (10 mM Hepes, 136 mM NaCl, 2.7 mM KCl, 2 mM MgCl_2_, 0.1% glucose, and 0.1% bovine serum albumin). Note that apyrase during the isolation procedure retained the platelet responses to ADP and ATP [12]. Platelet count was adjusted to 250 × 10^9^/L for aggregation and to 200 × 10^9^/L for cytosolic Ca^2+^ measurements.

### Light transmission aggregometry

Aggregation responses of washed platelets were measured with a Chronology aggregometer (Havertown PA, USA) at 37°C under stirring. The use of washed platelet suspensions in the presence of millimolar levels of extracellular Ca^2+^ and Mg^2+^allowed direct comparison with the [Ca^2+^]_i_ measurements, and furthermore prevented the need for recalcification of a citrate-anticoagulated PRP. The cells were pre- or post-treated with iloprost (10 nM), tirofiban (1 μg/mL) or PRT-060318 (5 μM) as indicated. For activation, sub-maximal concentrations were used of TRAP6 (10 μM) or CRP-XL (5 μg/mL). The PAR1 agonist instead of thrombin was used to prevent fibrin formation and factor XIIIa-dependent binding of fibrin to platelets [13]. Aggregation traces of % transmission (%T) were analysed for initial slope of the curves (Δ%T/min), area under the curve (%T, 10 min after agonist addition), and maximal response [14, 15]. See S1 Data.

### Cytosolic Ca^2+^ measurements

Pelleted platelets in Hepes buffer pH 7.45 were incubated with Fura-2 AM (3 μM) and pluronic (600 μM) in the presence of apyrase for 40 min at 37°C [16]. After a wash step, the Fura-2-loaded platelet were resuspended into Hepes buffer pH 7.45, and adjusted to 200 × 10^9^ platelets/L. For measurements of rises in cytosolic [Ca^2+^]_i_, the loaded cells in 96-wells plate were pre-incubated with sub-maximal concentrations of indicated (ant)agonist (TRAP6, CRP-XL, iloprost, Me-S-ADP, thrombin or vehicle solution) for 9 min [10]. Changes in fluorescence at 340 nm and 380 nm excitation per row over time were assessed using a FlexStation 3 robotic machine at 37°C, as described earlier [10]. After baseline fluorescence recording, CaCl_2_ (1 mM, f.c.) was added, followed by TRAP6 (10 μM, f.c.) or CRP-XL (5 μg/mL, f.c.) as a second agonist (1 min). Automated injection speed for agonist addition was set at 8 μL/s. Note that only a single agonist at a time could be automatically injected during the ratiometric fluorescence recording. After correction for background fluorescence per excitation wavelength, ratio fluorescence values were converted into levels of nanomolar levels of [Ca^2+^]_i_ using minimal and maximal ratio values from calibration wells with Triton-X-100 lysed platelets [16].

### Statistical analysis

Normally distributed data are presented as means ± SEM. Excel software was used for statistical analyses. Paired values were compared by a two-sided paired Student t-test, while unpaired values were compared by a two-sided 1-way ANOVA. Values of P < 0.05 were considered to be statistically significant.

## Results

### Prolonged activation of platelets after GPVI stimulation

**Fig 1:**
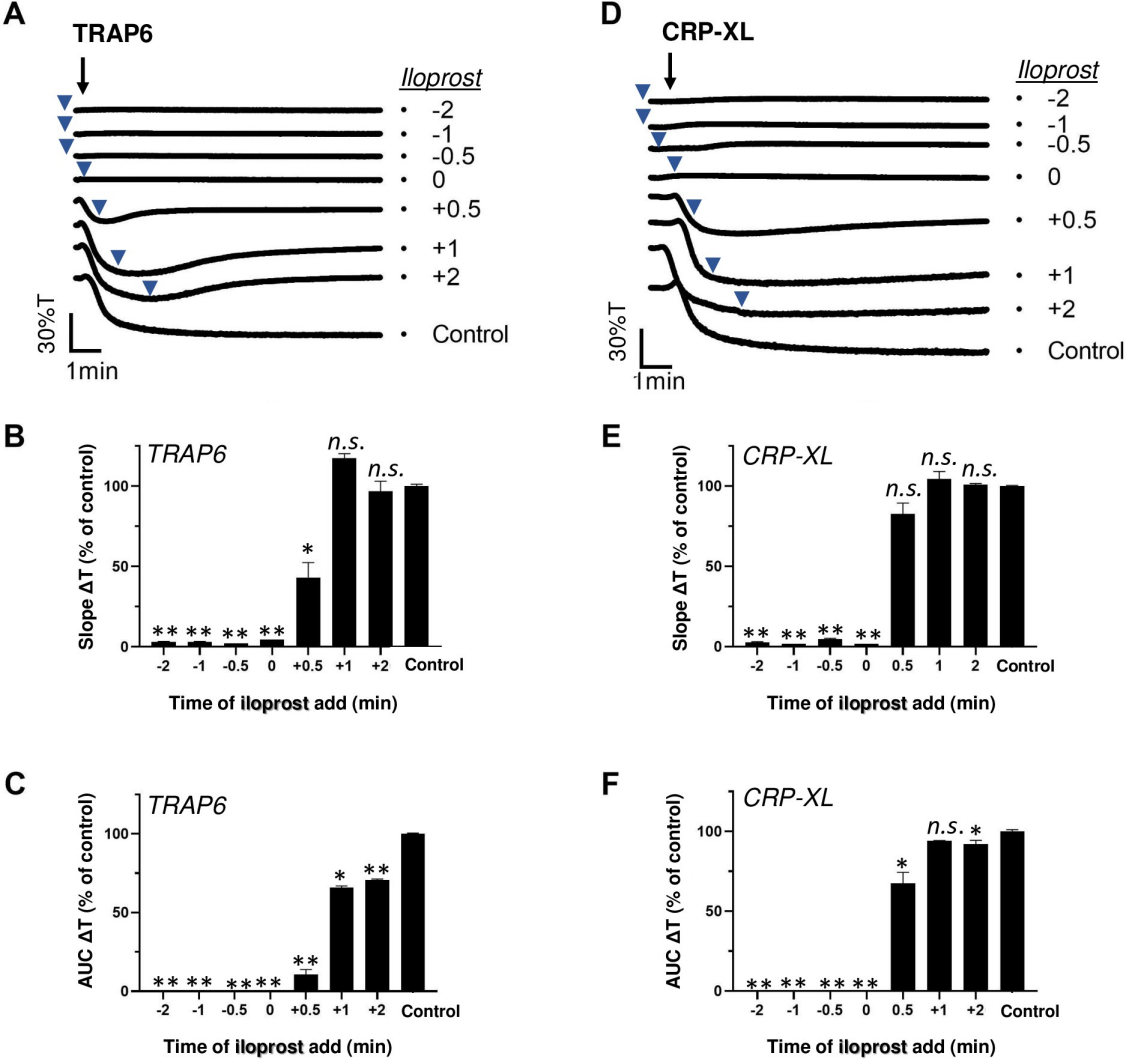
Post-hoc inhibitory effect of iloprost on TRAP6- and CRP-XL-induced platelet aggregation. Platelets in suspension were stimulated with 10 μM TRAP6 (A-C) or 5 μg/mL CRP-XL (D-F), and aggregation was recorded by light transmission aggregometry. Iloprost (10 nM) was added before (-2, -1 or -0.5 min), simultaneously with (0 min) or after (+0.5, +1, +2 min) the indicated agonist. Representative traces are shown); arrows indicate addition of agonist, arrowheads addition of iloprost (A, D). Graphs of aggregation slope as %T/min (B, E) and aggregation-area-under-the-curve (AUC, 10 min) (C, F), as fractions of control without iloprost. For maximal %T values, see S1 Data. Means ± SEM (n = 3). *P<0.05, **P<0.001 *vs*. control traces, paired Student t-test.

Platelet aggregation can be induced via the PAR1-type GPCR (for thrombin and TRAP6) or via the ITAM-linked receptor GPVI (for collagen and CRP-XL), where in either case pretreatment with the cAMP-elevating agent iloprost is known to suppress the aggregation process [1, 2]. However, it has remained unclear how long such a pretreatment needs to be for affecting the platelet responses. To investigate this, we added iloprost to human platelets at various times before or after stimulation using a submaximal dose of TRAP6 or CRP-XL. Markedly, the addition of iloprost, not only before but also simultaneously with TRAP6 or CRP-XL, completely annulled the aggregation responses (Fig 1A and 1D). Quantification of the aggregation traces pointed to abolition of the curve slope (Fig 1B and 1E) and the area-under-the-aggregation-curve (Fig 1C and 1F). Moreover, the inhibitory effect of iloprost retained up to 0.5–2 min after TRAP6 stimulation, in that it halted or partly reversed the initial aggregation process. However, this time frame of inhibition was shorter for up to 0.5 min in case of CRP-XL stimulation. These findings pointed to a more reduced ability of GPVI-stimulated platelets in terms of post-hoc responding to cAMP elevation.

**Fig 2:**
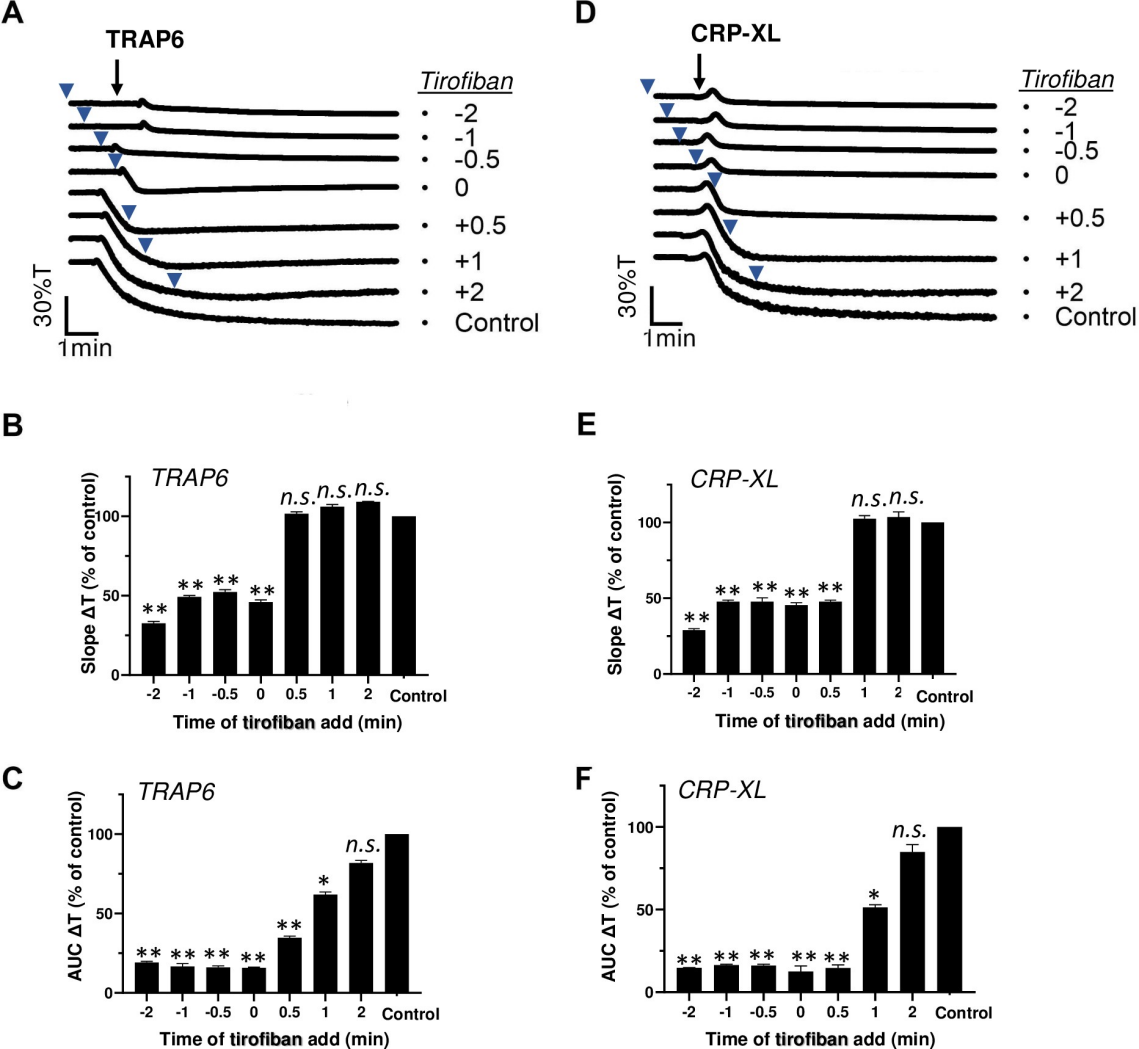
Post-hoc inhibitory effect of integrin antagonist on TRAP6- and CRP-XL-induced platelet aggregation. Platelets in suspension were stimulated with 10 μM TRAP6 (A-C) or 5 μg/mL CRP-XL (D-F), and aggregation was recorded by light transmission aggregometry. Tirofiban (1 μg/mL) was added before (-2, -1 or -0.5 min), simultaneously with (0 min) or after (+0.5, +1, +2 min) the indicated agonist. Representative traces are shown; arrows indicate addition of agonist, arrowheads addition of tirofiban (A, D). Bar graphs indicate the aggregation slope as %T/min (B, E) and aggregation-area-under-the-curve (AUC, 10 min) (C, F), as fractions of control without tirofiban. Means ± SEM (n = 3). *P<0.05, **P<0.001 *vs*. control traces, paired Student t-test.

Platelet aggregation relies on integrin α_IIb_β_3_ activation and fibrinogen-dependent platelet-platelet interactions [1]. To assess response versatility on the level of α_IIb_β_3_ activation, we performed similar experiments, in which we applied the integrin antagonist tirofiban before or after TRAP6 or CRP-XL. The tirofiban fully suppressed the aggregation response, when added before or together with either agonist (Fig 2A and 2D). Quantification of the traces learned that post-addition of tirofiban for up to 2 min did not influence the initial aggregation slope (Fig 2B and 2E), but partly affected the aggregation integral (Fig 2C and 2F) with TRAP6 or CRP-XL.

**Fig 3:**
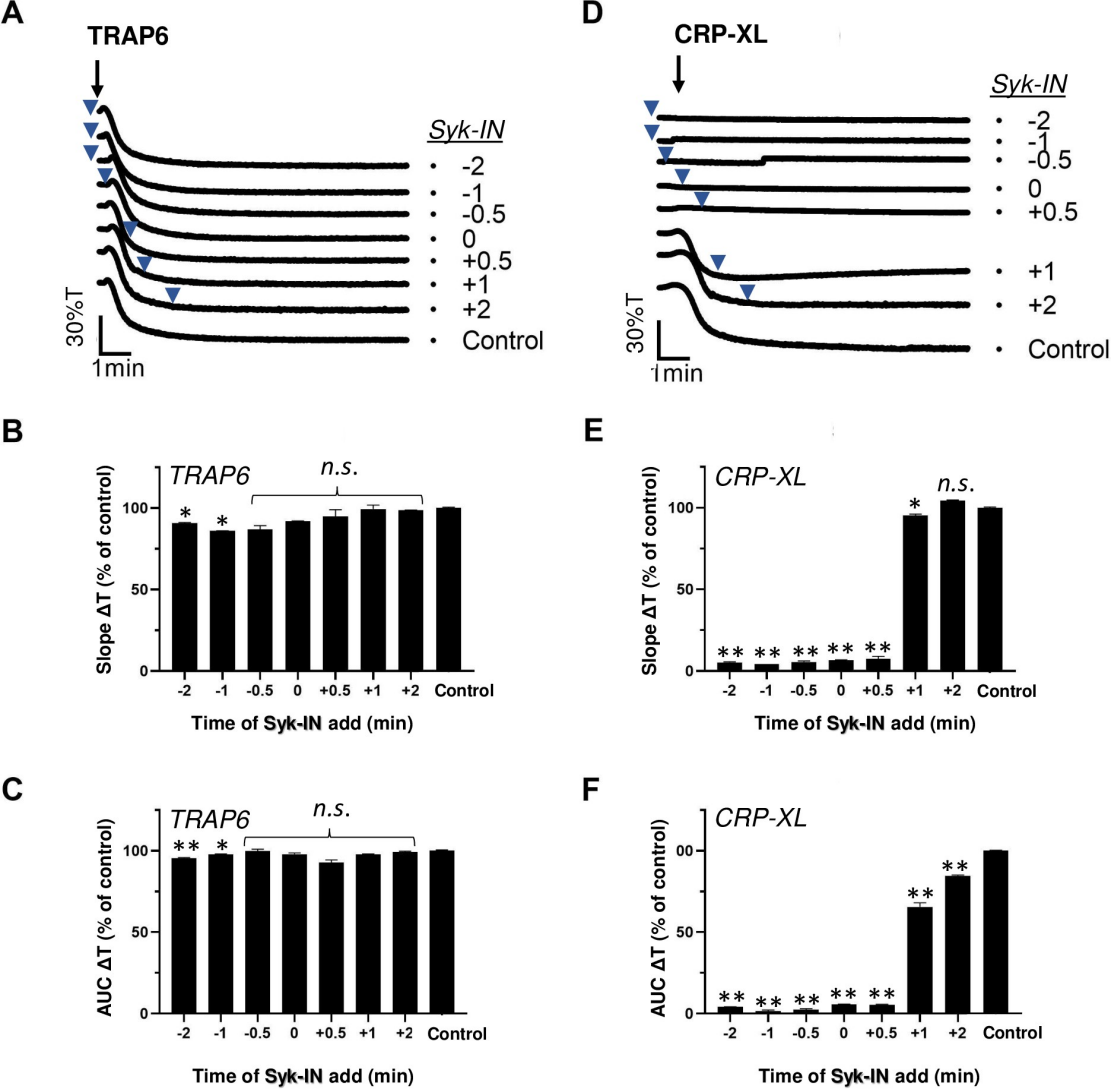
Post-hoc inhibitory effect of Syk blocker on CRP-XL-induced platelet aggregation. Platelets in suspension were stimulated with 10 μM TRAP6 (A-C) or 5 μg/mL CRP-XL (D-F), and aggregation was recorded by light transmission aggregometry. Syk-IN (5 μM) was added before (-2, -1 or -0.5 min), simultaneously with (0 min) or after (+0.5, +1, +2 min) the indicated agonist. Representative traces; arrows indicate addition of agonist, arrowheads addition of Syk-IN (A, D). Bar graphs of aggregation slope as%T/min (B, E) and aggregation-area-under-the-curve (AUC, 10 min) (C, F), expressed as fractions of control without Syk-IN. Means ± SEM (n = 3). *P<0.05, **P<0.001 *vs*. control traces, paired Student t-test.

Platelet activation induced by GPVI is known to rely on activation of the protein tyrosine kinase Syk [10]. To reveal the requirement of Syk signalling over time, we treated platelets with the selective inhibitor PRT-060318 (Syk-IN) [10] before or after stimulation by TRAP6 or CRP-XL. As expected, Syk-IN did hardly suppress the PAR1-mediated platelet aggregation at any time point (Fig 3A–3C). On the other hand, Syk-IN fully abrogated the GPVI-mediated aggregation, even when given after 0.5 min, *i*.*e*. when the aggregation normally would have started (Fig 3D–3F). At later time points up to 2 min, Syk-IN remained partly effective in suppressing the overall aggregation process (Fig 3F). This suggested that a relatively prolonged GPVI signal generation via Syk is required for completion of the aggregation process.

**Fig 4:**
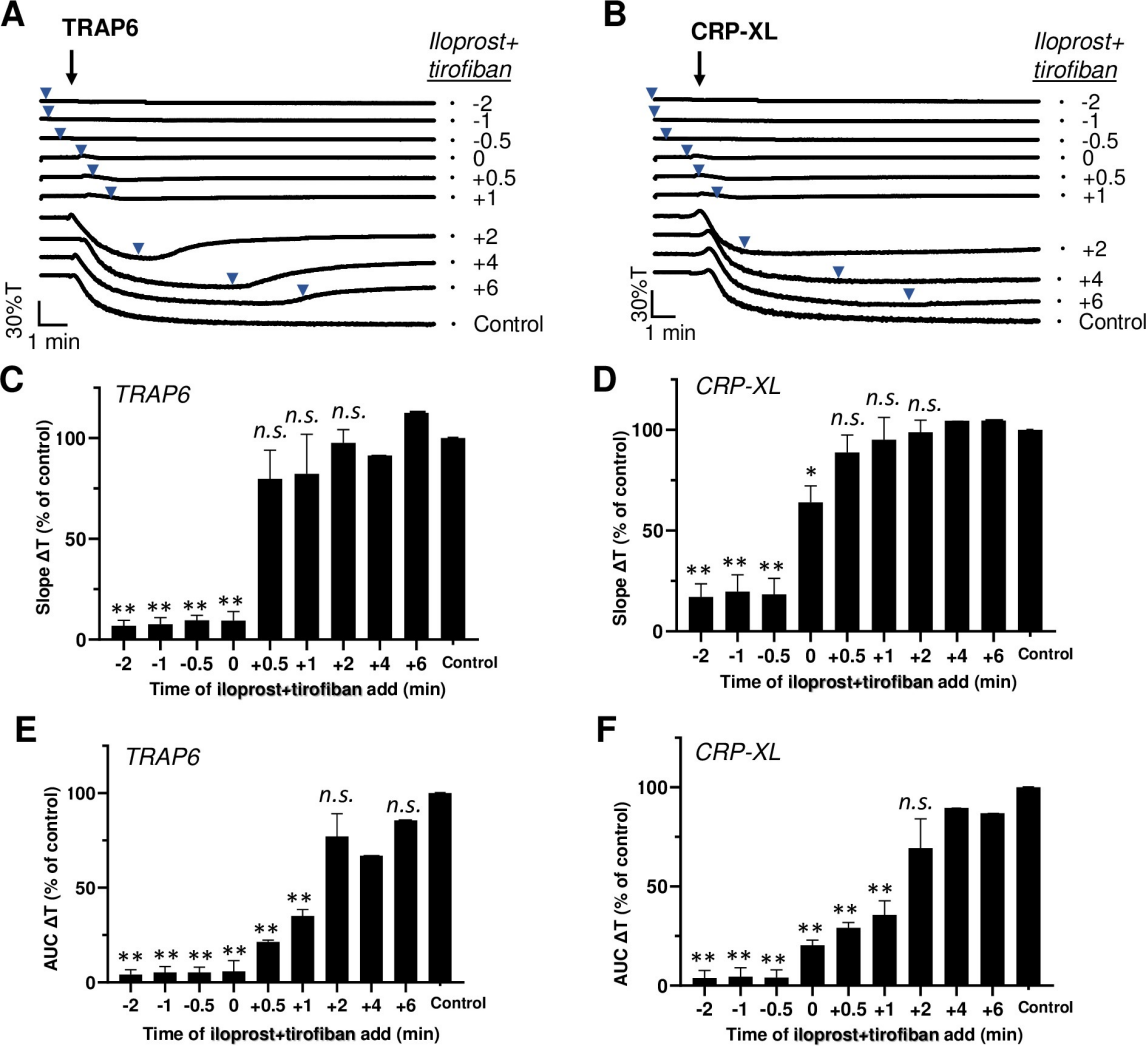
Post-hoc inhibitory effect of iloprost plus tirofiban on agonist-induced platelet aggregation. Platelets in suspension were stimulated with 10 μM TRAP6 (A-C) or 5 μg/mL CRP-XL (D-F), and aggregation was recorded by light transmission aggregometry. Iloprost (10 nM) in combination with tirofiban (1 μg/mL) was added before (-2, -1 or -0.5 min), simultaneously with (0 min) or after (+0.5, +1, +2, +4, +6 min) the indicated agonist. See Fig 1. Means ± SEM (n = 3). *P<0.05, **P<0.001 *vs*. control traces, paired Student t-test.

Given the ability of either iloprost or tirofiban to influence the aggregation after agonist addition, it was interesting to examine combined effects of the two inhibitors. When added before or up to 1 min after TRAP6 or CRP-XL stimulation, iloprost+tirofiban caused substantial suppression of the aggregation process (Fig 4A–4F). Typically, however, examination of the aggregation traces indicated that with TRAP6 even late application of the inhibitors (6 min) caused substantial disaggregation, while this was not the case with CRP-XL (Fig 4A and 4B). This again pointed to a more continued, less reversible activation signal with CRP-XL. By comparison, it appeared that iloprost postaddition after TRAP6 to a stronger extent than tirofiban postaddition (P<0.05) triggered the reversal of aggregation with 15–30% transmission.

In the presence of fibrinogen, iloprost on top of tirofiban also induced reversal of the aggregation response induced by Me-SADP (S1 Fig). Additional experiments indicated that, with supramaximal concentrations of TRAP6 (50 μM) or CRP-XL(25 μg/mL), the simultaneous application of iloprost+tirofiban still prevented the aggregation at *t* = 0, but not at *t* = 2 min, *i*.*e*. at a time point the aggregation process was completed (data not shown).

### More prolonged priming of Ca^2+^ signalling after GPVI stimulation in comparison to PAR stimulation

The results so far suggested an overall higher versatility of TRAP6- than of CRP-XL-induced aggregation in terms of secondary inhibition and response reversibility. To investigate how this extended to the intracellular signalling events, we measured the cytosolic [Ca^2+^]_i_ rises of platelets loaded with the probe Fura-2. Measurements were performed by 340/380 nm ratio fluorometry in 96-well plates, where an agonist could precisely be injected during the measurement time using a FlexStation 3 robot. This method results in diffusion-limited, but highly reproducible and calibrated [Ca^2+^]_i_ rises [10]. We developed an experimental setup, in which the platelets were manually treated with a first agonist or vehicle medium in wells, and then after a time window of 10 min were evaluated for changes in [Ca^2+^]_i_ using the FlexStation robot, which allowed to precisely record the signalling responses to the second agonist.

**Fig 5:**
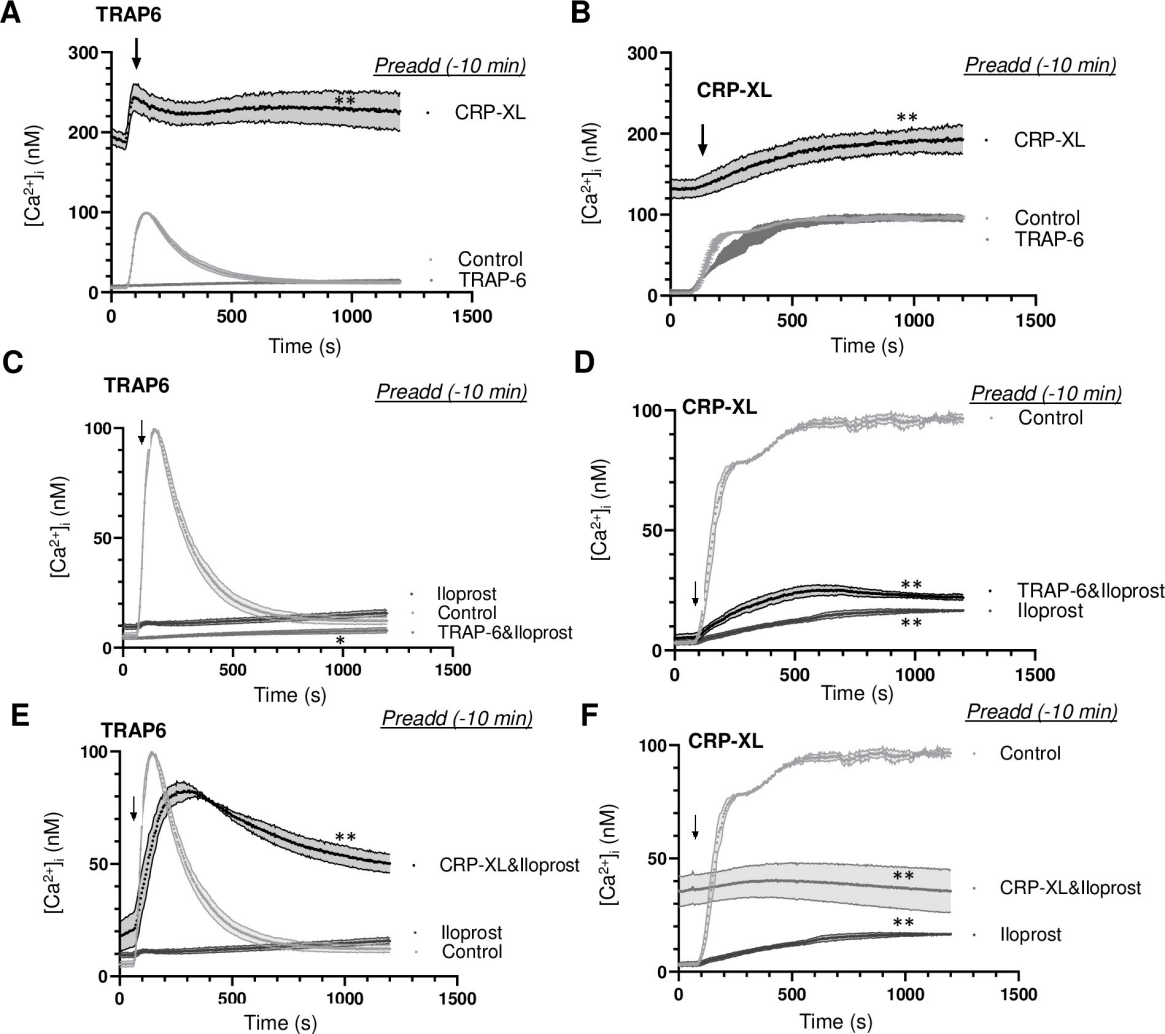
Recurrent Ca^2+^ signal generation induced by consecutive agonists. Calibrated [Ca^2+^]_i_ rises were recorded during 25 min of Fura-2-loaded platelets in 96-wells plates. Platelets were stimulated at indicated time point (arrow) as second agonist with 10 μM TRAP6 (A, C, E) or 5 μg/mL CRP-XL (B, D, F). (A, B) Preaddition of vehicle (control), TRAP6 (10 μM) or CRP-XL (5 μg/mL) at 10 min before second agonist. (C, D) Preaddition of vehicle control or iloprost (10 nM) with/without TRAP6 (10 μM) at 10 min before second agonist. (E, F) Preaddition of iloprost (10 nM) with/without CRP-XL (5 μg/mL) at 10 min before second agonist. Means ± SEM (n = 3 experiments). *P<0.05, **P<0.001 *vs*. controls at t = 1000 s, paired Student t-test.

In agreement with earlier findings [17], initial platelet stimulation with TRAP6 alone gave a fast and transient [Ca^2+^]_i_ rise (S2A Fig). Initial stimulation ith CRP-XP alone resulted in a slower and persistent [Ca^2+^]_i_ rise (S2B Fig). Markedly, after prior stimulation with CRP-XL, the subsequent application of TRAP6 again showed the transient [Ca^2+^]_i_ rise on top of the CRP-XL signal (Fig 5A). In constrast, prior stimulation with TRAP6 completely annuled the response to a second TRAP6 addition. In contrast, after initial stimulation with TRAP6 or CRP-XL, the subsequent addition of CRP-XL resulted in another prolonged Ca^2+^ signal (Fig 5B). This pointed to a relatively prolonged priming effect of the initial CRP-XL stimulation in comparison to initial PAR1 stimulation.

As expected, pre-incubation with iloprost (10 min) completely annulled the [Ca^2+^]_i_ rises evoked by TRAP6 (Fig 5C) or by CRP-XL (Fig 5D). However, after iloprost plus TRAP6, the cells responded to later CRP-XL by a greatly reduced [Ca^2+^]_i_ rise, pointing to a residual suppression by iloprost (Fig 5D). On the other hand, after iloprost plus CRP-XL, the platelets responded to later TRAP6 by a prolonged Ca^2+^ signal (Fig 5E), reminiscent of the normal CRP-XL response. Suppression by iloprost was also observed upon dual addition of TRAP6 (Fig 5C). With iloprost present, dual addition of CRP-XL resulted in a slightly increased [Ca^2+^]_i_ response (Fig 5F). Together, these data showed that, also in the presence of iloprost, initial CRP-XL stimulation enhanced the Ca^2+^ responses of later TRAP6 stimulation.

**Fig 6:**
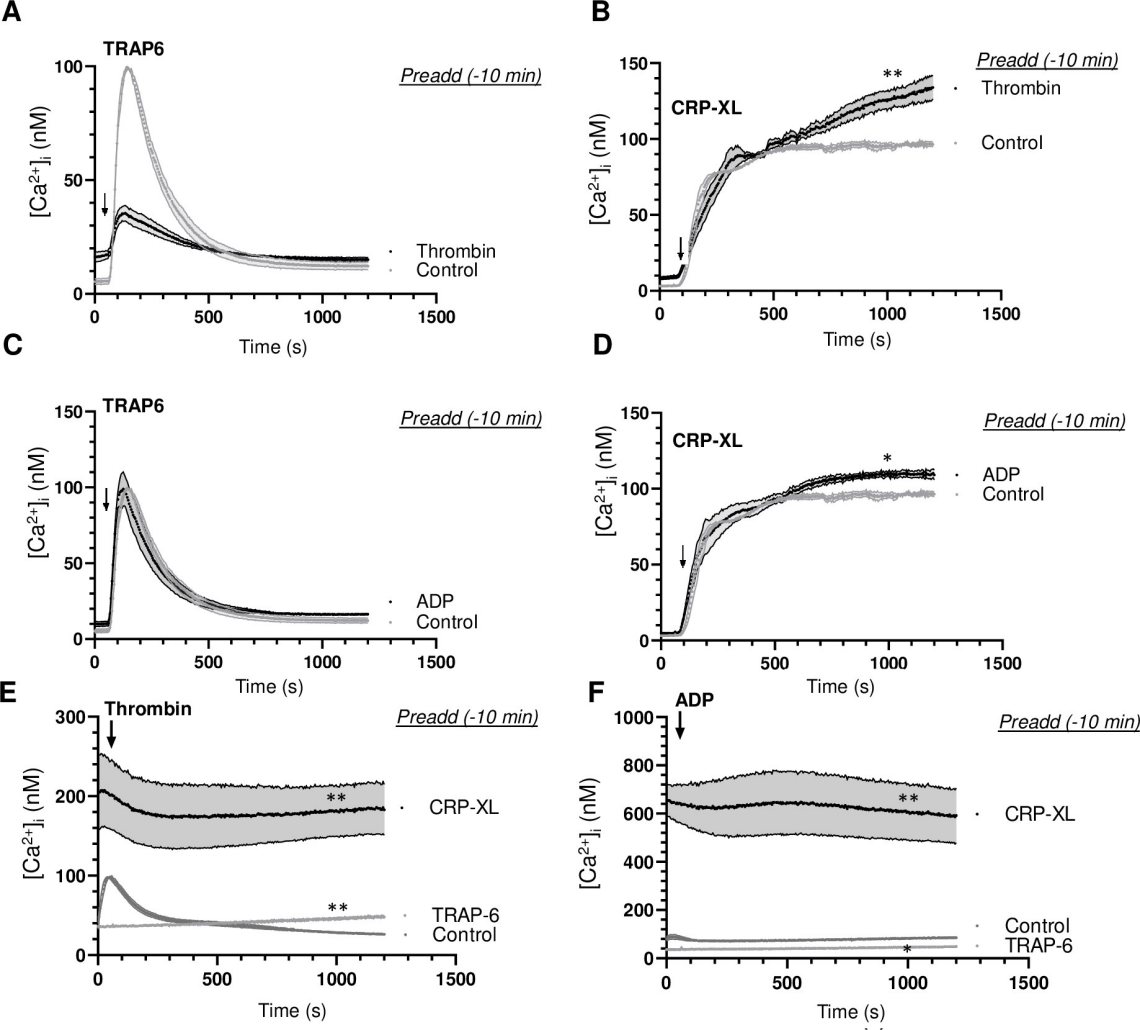
Effect of agonist stimulation on Ca^2+^ signalling induced by a second agonist. Calibrated [Ca^2+^]_i_ rises were recorded during 25 min of Fura-2-loaded platelets in 96-wells plates. Platelets were stimulated at indicated time point (arrow) as second agonist with 10 μM TRAP6 (A, C), 5 μg/mL CRP-XL (B, D), 1 nM thrombin (E) or 1 μM Me-S-ADP (F). (A, B) Preaddition of vehicle (control) or thrombin (1 nM) at 10 min before second agonist. (C, D) Preaddition of vehicle (control) or Me-S-ADP (1 μM) at 10 min before second agonist. (E, F) Preaddition of vehicle (control), TRAP6 (10 μM) or CRP-XL (5 μg/mL) at 10 min before second agonist. Means ± SEM (n = 3 experiments). *P<0.05, **P<0.001 *vs*. controls at *t* = 1000 s, paired Student t-test.

To determine if such a priming effect was confined to prestimulation with CRP-XL, we performed similar experiments with thrombin or ADP as a first agonist (t = -10 min). Herein, prior thrombin impaired the TRAP6-induced [Ca^2+^]_i_ rise (Fig 6A), whilst prior ADP was without effect (Fig 6C). On the other hand, prior thrombin (Fig 6B) but not ADP (Fig 6D) enhanced the [Ca^2+^]_i_ rise induced by CRP-XL. Prior TRAP6 almost completely abolished the respond to secondary thrombin or ADP (Fig 6E and 6F). Markedly, however, prior CRP-XL caused a prolonged and high Ca^2+^ signal, while the platelets still responded to thrombin or ADP (Fig 6E and 6F). This indicated that PAR stimulation (with TRAP6 or thrombin) protected against secondary stimulation of these receptors. Taken together we concluded that, in terms of Ca^2+^ signalling, prior GPVI (CRP-XL) but not PAR stimulation provoked an prolonged, high activation state, which still allowed the platelets to respond to a second GPCR agonist, such as thrombin, TRAP6 or ADP.

## Discussion

In this paper, we present new proof-of-concept data that iloprost (cAMP elevation), tirofiban (integrin α_IIb_β_3_ blocker) and/or Syk kinase inhibition (sub)acutely affect the starting or ongoing platelet aggregation process in response to TRAP6 or CRP-XL, even when applied after these agonists. However, when compared to PAR stimulation with TRAP6, GPVI stimulation with CRP-XL provided a shorter time window for the aggregation process to stop upon secondary application of iloprost. Subacute inhibition of the tyrosine kinase Syk was only effective in case of GPVI stimulation. This pointed to a more continued, less reversible activation signal with CRP-XL. In agreement with this, also the [Ca^2+^]_i_ measurements showed a more persistent activation state of the platelets after GPVI stimulation than after PAR1 stimulation, thus influencing the platelet responses to a second agonist.

Interestingly, it appeared that the platelets after initial PAR1 stimulation retained their responsiveness to GPVI stimulation, but became insensitive to a recurrent stimulation of the PAR1 receptor. Overall, tis work disclosed an unexpected high versatility of activated platelets in their ability to acutely respond to a subsequent receptor agonist, with a more prolonged signal memory effect after GPVI than after PAR stimulation.

Iloprost as a prostacyclin mimetic has been used in the clinic for platelet inhibition and vasodilatation in pulmonary hypertension treatment [18]. Our aggregation experiments indicate that late application of this inhibitor can reverse the platelet activation process even after initial receptor stimulation, yet in a way depending on the agonist and receptor type. Typically, the post-hoc inhibitory effect of iloprost decreased in the order of TRAP6 > CRP-XL. In combination with integrin α_IIb_β_3_ antagonist tirofiban, iloprost acted even by causing substantial disaggregation after TRAP6, but not CRP-XL stimulation.

In agreement with our findings, it is known that iloprost pretreatment strongly affects TRAP6-induced platelet responses [19]. Iloprost, acting on the level of G-proteins (Gs) via adenylyl cyclase, directly competes with soluble GPCR agonists (thrombin, ADP, TxA_2_) also acting on adenylyl cyclase via Gi [20–22]. Hence, the joint regulation of adenylyl cyclase activity can result in a versatile modulation of cAMP levels and PKA activity. Earlier, we have established that iloprost and ADP in terms of PKA-dependent phosphorylation act partly in an antagonistic way [23]. Several of the PKA-regulated phosphorylation sites are cytoskeletal proteins, which agrees with the notion that platelet disaggregation is accompanied by a reversal of cytoskeletal changes [24].

Mechanistically, both the prior and subactue effects of iloprost will be due to an immediate elevation in cAMP and protein kinase A (PKA), which then suppresses phospholipase C activity and ensuing [Ca^2+^]_i_ rises [25–27]. The more limited ability of iloprost to revert the CRP-XL induced aggregation (in comparison to TRAP6) can be explained by the longer Ca^2+^ signal with this GPVI agonist, implying a lower ability of PKA to overrule this signal. On the other hand, our laboratories have described that iloprost and other cAMP-elevating agents, as a pretreatement, are able to affect the collagen-induced platelet adhesion under stasis and flow conditions [28, 29].

Tirofiban as an integrin antagonist is in use for the prevention of thrombus formation in ischemic stroke [30]. The observed reversal of TRAP6-induced aggregation by tirofiban/iloprost is compatible with evidence from preclinical studies that integrin activation per se is a reversible process [1, 31]. The acute halting of the CRP-XL-induced aggregation with inhibitor Syk-IN (with antithrombotic potential in mouse) is explained by the evidence that it is an upstream blocker of GPVI- and CLEC2-induced signalling pathways [10, 32]. Markedly, the blockage of Syk activation (Syk-IN) or of integrin α_IIb_β_3_ activation (tirofiban) primarily stopped the ongoing aggregation process without reversal. It thus appears that interference at different levels of the signalling scheme (early Syk activation, or late integrin activation) can halt the further assembly of platelet aggregates, but cannot disassemble the aggregates, such as is the case with the PKA-activating compound iloprost.

The [Ca^2+^]_i_ measurements provided proof-of-principle evidence that platelets have the ability to respond consecutively to PAR1, P2Y and GPVI stimulation, even when these receptors are triggered 10 min apart. Interestingly, such sequential responses were not seen for dual exposure to PAR1 agonist (TRAP6, thrombin), in that a first stimulation inactivated the Ca^2+^ signal of a second stimulation. This points to a mechanism of thrombin receptor desensitisation after platelet exposure to PAR agonists [33, 34]. It also underlines the idea that platelet exposure to a gradient of GPCR agonists causes response diminishment [35].

Whilst it is known that continued receptor occupancy is needed for the completion of thrombin-induced [Ca^2+^]_i_ rises [17, 36], we now find that—on the longer term (10–30 min) -, PAR1-stimulated platelets in some way become reset by a desensitisation mechanism. Interestingly, such a desensitisation process does not occur following GPVI stimulation, hence excluding a role of cleavage of the GPVI receptors [37]. Rather, the maintained Ca^2+^ signal with CRP-XL points to a longer-term high activation state (priming) of the platelets, which can be linked to the prolonged ability of store-regulated Ca^2+^ entry with GPVI ligands [38].

Regarding the translation relevance of our findings, at the one hand, we show that platelets have the ability to respond to multiple agonists, for instance when (in the circulation) exposed to soluble agonists or when adhered to subendothelial vascular agonists like collagen. At the other hand, our data provide more insight into the mechanism of positive and (secondarily) negative platelet priming by dual exposure to agonists and/or antagonists. More precisely, our findings suggest that the acute exposure of circulating platelets to locally generated thrombin (before it is inactivated by antithrombin) results in an only shortly increased activation state, which is antagonised by the simultaneous exposure to prostacyclin. On the other hand, it appears that platelet adhesion to vascular collagen resulting in a GPVI-mediated aggregation leads to a more persistent activation state, which influences the ensuing responses to a next agent.

Summarising, we provide novel data that GPVI stimulation causes a more prolonged and robust priming and memory activating effect on platelets, when compared to PAR or P2Y stimulation. This work thereby revealed unexpected differences between a high versatility of platelets in sequentially responding to specific agonists and antagonists.

## Supporting information

S1 Fig Post-hoc inhibitory effect of iloprost and/or tirofiban on agonist-induced platelet aggregation in the presence of fibrinogen. (DOCX)

S2 Fig Calcium responses by one agonist. (DOCX)

S1 Data (XLSX)
