# Supplementary material for: Long-term platelet priming after glycoprotein VI stimulation in comparison to Protease-Activating Receptor (PAR) stimulation
Source: PLoS One. 2021 Mar 3;16(3):e0247425. doi: 10.1371/journal.pone.0247425 (PMC7928515; doi:10.1371/journal.pone.0247425)
Supplement: S1 Fig — (DOCX) [file pone.0247425.s001.docx]

**S1 Fig. Post-hoc inhibitory effect of iloprost and/or tirofiban on agonist-induced platelet aggregation in the presence of fibrinogen.**

Platelets in suspension containing 50 µg/mL fibrinogen were stimulated with 1 µM Me-S-ADP (**A-D**) or 10 µM TRAP6 (**E, F**), and aggregation was recorded by light transmission aggregometry. Iloprost (10 nM) and/or tirofiban (1 µg/mL) was added before (-2 min), simultaneously with (0 min) or after (+0.5, +1, +2 min) the indicated agonist. Shown are representative aggregation traces (**A**, **C**, **E**). Arrowheads indicate addition of iloprost and/or tirofiban. Bar graphs indicate the aggregation-area-under-the-curve (AUC, 10 min), as fractions of control (**B**, **D**, **F**). See further Fig 1. Means ± SEM (n = 3). **P<0.001 *vs.* control traces, paired Student t-test.
