# Supplementary material for: Long-term platelet priming after glycoprotein VI stimulation in comparison to Protease-Activating Receptor (PAR) stimulation
Source: PLoS One. 2021 Mar 3;16(3):e0247425. doi: 10.1371/journal.pone.0247425 (PMC7928515; doi:10.1371/journal.pone.0247425)
Supplement: S2 Fig — (DOCX) [file pone.0247425.s002.docx]

**S2 Fig.** **Calcium responses by one agonist.**

Cytosolic Ca^2+^ rises were recorded of Fura-2-loaded platelets in 96-wells plates during 33 min. (A) Cells were preincubated with vehicle (control), tirofiban (1 µg/mL) or Syk-IN (5 µM). The platelets were stimulated with 10 µM TRAP6 (**A**) or 5 µg/mL CRP-XL (**B**) during fluorescence recording. Means ± SEM (n = 3 experiments).
